# Sulfur Compounds as Inhibitors of Enzymatic Activity of a Snake Venom Phospholipase A_2_: Benzyl 4-nitrobenzenecarbodithioate as a Case of Study

**DOI:** 10.3390/molecules25061373

**Published:** 2020-03-18

**Authors:** Isabel Henao Castañeda, Jaime Andrés Pereañez, Lina María Preciado, Jorge Jios

**Affiliations:** 1 Grupo de Investigación en Productos Naturales Marinos, Departamento de Farmacia, Facultad de Ciencias Farmacéuticas y Alimentarias, Universidad de Antioquia UdeA, Calle 70 No. 52–21, 050010 Medellín, Colombia; 2 Programa de Ofidismo/Escorpionismo, Departamento de Farmacia, Facultad de Ciencias Farmacéuticas y Alimentarias, Universidad de Antioquia UdeA, Calle 70 No. 52–21, 050010 Medellín, Colombia.; andrespj20@gmail.com (J.A.P.); linampr@gmail.com (L.M.P.); 3 Laboratorio UPL (Unidad PLAPIMU-LASEISIC), Campus Tecnológico Gonnet (CIC-BA), Cno. Centenario e/505 y 508, 1897 Gonnet, Argentina; jljios@quimica.unlp.edu.ar; 4 Departamento de Química, Facultad de Ciencias Exactas, Universidad Nacional de La Plata, 47 esq. 115, 1900 La Plata, Argentina

**Keywords:** PLA_2_, Inhibitor, Thioester, Carbodithioate, Molecular Docking, Snake venoms

## Abstract

Snakebite is a neglected disease with a high impact in tropical and subtropical countries. Therapy based on antivenom has limited efficacy in local tissue damage caused by venoms. Phospholipases A_2_ (PLA_2_) are enzymes that abundantly occur in snake venoms and induce several systemic and local effects. Furthermore, sulfur compounds such as thioesters have an inhibitory capacity against a snake venom PLA_2_. Hence, the objective of this work was to obtain a carbodithioate from a thioester with known activity against PLA_2_ and test its ability to inhibit the same enzyme. Benzyl 4-nitrobenzenecarbodithioate (I) was synthesized, purified, and characterized using as precursor 4-nitrothiobenzoic acid S-benzyl ester (II). Compound I showed inhibition of the enzymatic activity a PLA_2_ isolated from the venom of the Colombian rattlesnake *Crotalus durissus cumanensis* with an IC_50_ of 55.58 μM. This result is comparable with the reported inhibition obtained for II. Computational calculations were performed to support the study, and molecular docking results suggested that compounds I and II interact with the active site residues of the enzyme, impeding the normal catalysis cycle and attachment of the substrate to the active site of the PLA_2_.

## 1. Introduction

Based on our previous results, we decided to compare the biological activity of a thioester with the corresponding carbodithioate exchanging oxygen by sulfur. We studied the ability of benzyl 4-nitrobenzenecarbodithioate (I) to inhibit a myotoxic Asp-49 phospholipases A_2_ (PLA_2_) from *Crotalus durissus cumanensis* venom, as we are interested in the search for inhibitors of snake venom enzymes that complement antivenom therapy. In this work, we show, for the first time, the capacity of a carbodithioate to inhibit snake venom toxins.

The World Health Organization (WHO) recognizes snakebite as a Neglected Tropical Disease [1]. Each year between 4.5 and 5.4 million people are bitten by snakes [2], mainly poorer people living in rural areas of tropical countries; hence, this disease has significant medical importance since it is associated with high morbidity and mortality. There are between 81,410 and 137,880 deaths per year, and approximately three times this number of people are left with permanent disabilities [3]. In 2018, the Colombian National Institute of Health reported 5434 ophidian accidents [4].

Systemic and local effects of snakebite envenomations are caused by the action of several enzymes, proteins, and peptides, including phospholipases A_2_ (PLA_2_) [5]. PLA_2_ from Viperidae venoms are myotoxins that cause local and systemic myotoxicity and myonecrosis via the disruption of the plasma membrane [6,7]. PLA_2_ are one of the most abundant muscle-damaging components present in snake venoms. Among them, myotoxic PLA_2_ are calcium-dependent enzymes that catalyze the breakdown of glycerophospholipids, the main component of biological membranes, into lysophospholipids. The latter is responsible for the release of fatty acids, such as arachidonic acid, which is a precursor of pro-inflammatory eicosanoids [7]. Moreover, such degradation can lead to destabilization of the membrane bilayer [8].

Usually, PLA_2_ have 119 to 134 residues, three long helixes, two antiparallel β sheets, and a calcium-binding loop. The hydrophobic channel is composed of the antiparallel α helixes (residues 37–57 and 90–109, respectively) together with the N-terminal helix (residues 1–12). This structure leads the substrate to the active site formed by His48, Asp49, Tyr52, and Asp99. Additionally, Asp49, Tyr28, Gly30, and Gly32 form the calcium-binding loop that coordinates the Ca^2+^ and helps in the stabilization of the tetrahedral intermediate during catalysis [8]. Furthermore, there is an interfacial binding surface that allows the lipid–water interface of the phospholipid membrane bilayer.

Viperidae snake venoms also contain catalytically inactive PLA_2_ homologues, which have some changes in their primary structure, highlighting a specific mutation in the catalytic residue that changes Asp49 by Lys, Ser, Arg, Asn or Gln. Nevertheless, these PLA_2_ also induce myotoxicity and edema, by a mechanism independent of enzymatic hydrolysis of membrane glycerophospholipids. These toxins require an allosteric activation induced by the binding of fatty acid into the PLA_2_ hydrophobic channel, which activates specific sites for docking and disrupting membrane glycerophospholipids. Finally, destabilization occurs, and myotoxicity takes place [9].

Currently, there are no alternative or complementary therapies to antivenom for snakebite envenoming [10], although a considerable number of reports demonstrated a limited efficacy of the actual therapy against the local tissue damage [11]. This justifies the efforts of many research groups to find new molecules capable of inhibiting the effect of enzymes that cause local tissue damage and could act as a complement to antivenom therapy.

The search for new inhibitors of snake venom enzymes has become a topic of growing interest. There are reports of PLA_2_ inhibitors from natural sources and synthesis, including substituted thiobenzoic acid S-benzyl esters [12], naringenin derivatives [13], Aiplai, a compound from *Azadirachta indica* [14], cholic and ursodeoxycholic acids [15]. Varespladib, a pharmacological drug initially designed to inhibit mammal PLA_2_, recently proved effective in inhibiting snake venom PLA_2_, including *Bothrops asper* and *Bothrops jararaca* with IC_50_ of 0.0001 and 0.0002 μM [16]. Some compounds have reported IC_50_ with the same methodology used in this work, namely Pinostrobin, a flavonone isolated from *Renealmia alpinia* (IC_50_ of 1.85 mM) [17], Morelloflavone, a biflavonoid from *Garcinia madruno*, with IC_50_ of 0.38 mM [18], and thioesters derived from 2-sulfenyl ethylacetate, with IC_50_s between 132.7 and 305.4 μM [19].

**Figure 1:**
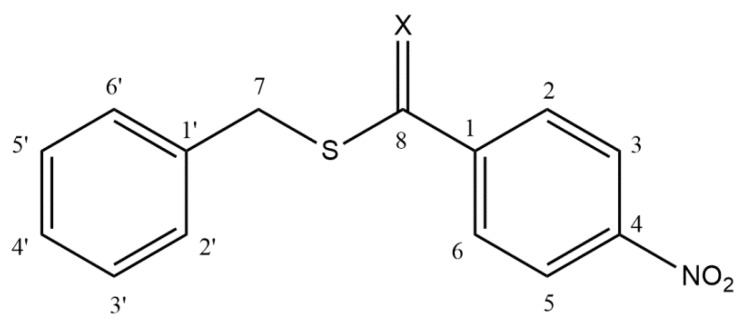
General structure of studied compounds: Benzyl 4-nitrobenzenecarbodithioate (**I**) and 4-nitrothiobenzoic acid S-benzyl ester (**II**).

In previous studies, we found that substituted thiobenzoic acid S-benzyl esters and thioesters derived from 2-sulfenyl ethylacetate are inhibitors of an Asp49-PLA_2_ enzyme isolated from the venom of the Colombian *Crotalus durissus cumanensis* rattlesnake in micromolar concentrations [12,19]. In this study, the title compound belongs to the carbodithioates, R-C(S)-S-R’, a family closely related to thioesters R-C(O)-S-R’. Changing the carbonyl C(O) function to thiocarbonyl C(S) implies some structural differences that deserve to be mentioned. In comparison to the corresponding C=O bond, a lengthening of the C-S interatomic distance is expected due to the ineffective overlapping between p-orbitals of the first (C) and second (S) row atoms. It transforms the π bond of the C=S group into C-S; therefore, a more diffuse charge transfer toward the bulky and slightly more electronegative sulfur atom C+-S- is expected. These hypotheses prompted us to determine the PLA_2_ inhibition of the tittle carbodithioate I. The structures of I and the related thioester II (4-nitrothiobenzoic acid S-benzyl ester, previous work) are shown in Figure 1, and some of their calculated physicochemical properties are found in Table 1. Both compounds have the same syn/anti planar conformational preferences around the ψ(X-C8-S-C7) torsion angle [20,21]. The X-ray structure of I [22] shows a syn conformation for the carbodithioate moiety with a planar geometry [ψ(S=C8-S-C7) = -1.2°].

Several methods to synthesize carbodithioates are described in the literature, and the oxygen/sulfur exchange is one of the simplest and direct ways to convert a thioester in carbodithioate compound. Although the Lawesson reagent [23,24] is widely used for this purpose, 2,4-Bis(4-phenoxyphenyl)-1,3,2,4-dithiadiphsophetane 2,4-disulfide was found more useful and effective for obtaining I [25].

Compound I was prepared in order to test its inhibitory ability on the enzymatic activity of a snake venom PLA_2_. Theoretical calculations were also performed to determine optimized geometries and vibrational frequencies to assist the experimental infrared band assignments. Moreover, experimental parameters obtained from the X-ray crystal structure of I [22] were used to validate the theoretical results.

Finally, a molecular docking study of I and II was carried out to suggest a mode of action of both compounds to evaluate the influence of the carbonyl and thiocarbonyl groups on the affinity for the active site of PLA_2_. 

## 2. Results

### 2.1. Conformational Analysis

The experimental structural parameters of I were taken from the single-crystal X-ray diffraction results [22]. The carbodithioate function shows a syn conformation with the chain S=C8-S-C7-C1′ within this plane with dihedral angles ψ(S=C8-S-C7) = −1.2° and ψ(C8-S-C7-C1′) = −174.7°. The phenyl and nitrophenyl rings are strongly deviated [ψ(S-C7-C1′-C2′) = 62.9°] and almost coplanar [ψ(S=C8-C1-C2) = 7.7°], respectively, from this plane. The interatomic distances involving the sulfur atoms in the thiocarbonyl and thioether C8-S-C7 groups are 1.644(3)Å (C8=S), 1.714(3)Å (C8-S), and 1.807(4)Å (C7-S).

The calculated parameters were obtained by B3LYP/6-31+G(d,p) level of approximation using Gaussian 09 (Gaussian Inc, Wallingford, CT, USA) [26] program package. The optimized free molecule represents the global minimum having no negative frequencies. The calculated potential energy curve for the torsion around the dihedral angle ψ(S=C8-S-C7) showed two minima for the synperiplanar and +anticlinal conformations, being the +anticlinal 28 kJ/mol higher in energy than the synperiplanar form. The last conformation was used to perform the vibrational frequencies calculations. The bond distances and angles were compared with the experimental ones (single crystal X-ray diffraction spectroscopy) and are shown in Table S1 (Supplementary Material). The carbodithioate function connecting both aromatic rings is almost planar (ψ(S=C8-S-C7) = 2.65°, ψ(C8-S-C7-C1′) = 179.01°), but the phenyl and nitrophenyl moieties are twisted in respect to this plane in 91.42° and 36.25°, respectively. The bond distances C8=S, C8-S, and C7-S, are 1.653 Å, 1.758 Å, and 1.847 Å, respectively.

### 2.2. Vibrational Analysis

Compound I has 84 normal vibrational modes. The experimental, most representative IR bands are shown in Table 2. The C=S stretching is a very strong band at 1048 cm^−1^ and 1065 cm^−1^ in the experimental and calculated spectra, respectively. The antisymmetric stretching for S-C8-C1 involves the carbodithioate moiety and is observed at 895 cm^−1^ (experimental) and 902 cm^−1^ (calculated). For the nitro group, two bands corresponding to the antisymmetric and symmetric stretching vibration have been measured at 1516 and 1343 cm^−1^, respectively (calculated: 1589 and 1382 cm^−1^).

### 2.3. Biological Activity 

**Figure 2:**
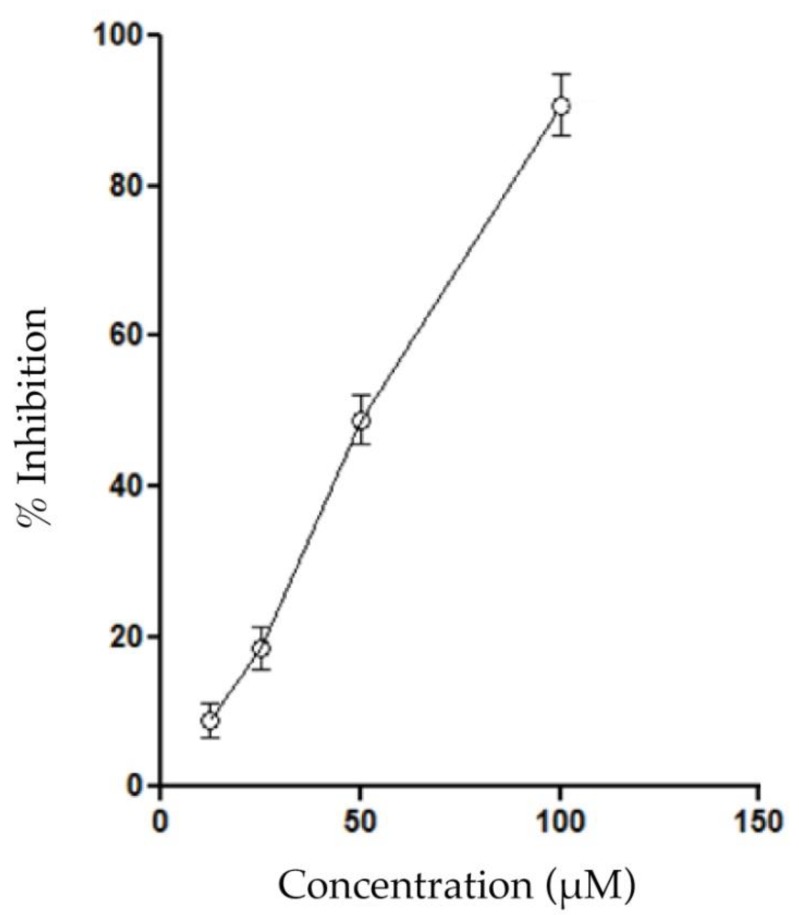
Inhibition of phospholipases A_2_ (PLA_2_) activity by I. Different concentrations were pre-incubated with 20 μg of PLA_2._ Results are shown as mean ± SEM, *n* = 6.

The carbodithioate I inhibited the enzymatic activity of a myotoxic PLA_2_ from *Crotalus durissus cumanensis* venom (Colombian rattlesnake) with an IC_50_ of 55.58 ± 4.6 μM, which was calculated from the linear portion of the dose-response curve (Figure 2). The inhibitory capacity of I is comparable with the reported inhibition for the thioester II (52% of inhibition at a concentration of 50 μM) [12].

### 2.4. Molecular Docking Study of I and II

To understand the mechanism of inhibition and compare the interactions of PLA_2_ with compounds I and II, we performed a molecular docking study using the available protein structure (PDB ID 2QOG).

**Figure 3:**
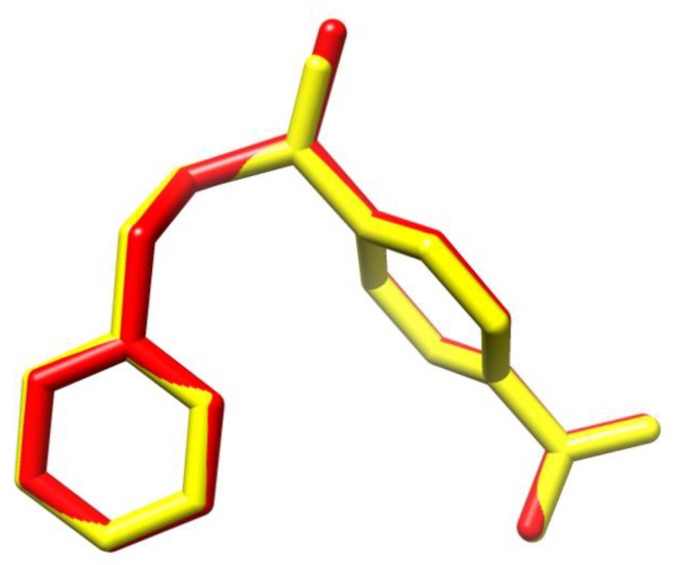
Docking conformations with the highest affinities for compounds **I** (red) and **II** (yellow).

Docking conformations with the lowest binding energy (−7.1 kcal/mol) for both ligands are shown in Figure 3. It is observed that these conformations are very similar and are almost entirely aligned. As expected, the most significant difference is observed in the distances of (C=S) and (C=O) bonds with 1.65 Å and 1.22 Å, respectively. 

**Figure 4:**
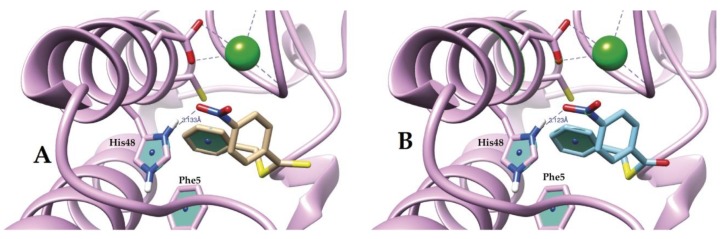
Hydrogen bond PLA_2_ with compounds I (**A**), II (**B**).

Additionally, we found hydrogen bonds with Nδ1 of His48 as the donor, and oxygen from the nitro group as the acceptor, with distances of 3.133 Å and 3.123 Å for ligands I and II, respectively (Figure 4). 

Furthermore, docking results suggested a π-π T-shaped interaction between Phe5 and the benzyl ring. The distances among the centroids of the aromatic rings involved in the interactions were 4.545 Å and 4.528 Å for I and II, respectively. The angles within the aromatic ring planes were 75.9° and 73.5°.

**Figure 5:**
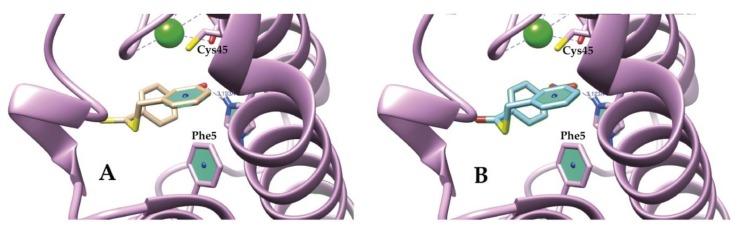
Benzyl ring contacts of I (**A**) and II (**B**) with Phe5 (π-π T shaped interaction) and with the sulfur atom of Cys45 (π-sulfur interaction).

On the opposite face of the benzyl ring, the ligands showed a π-sulfur interaction with Cys45. The distances between the centroids of each benzyl ring and the sulfur atom of Cys45 were 3.409 Å for both compounds (Figure 5).

Furthermore, a π-π T-shaped interaction was found between the imidazole ring of His48 and the benzyl ring of the studied compounds. The distances were 4.985 Å and 5.017 Å, and the angles were 77.8° and 75.4° for I and II, respectively.

Docking results suggested several Van der Waals interactions between ligands I and II and amino acids in the active site, the hydrophobic channel, and the interfacial binding surface of the enzyme.

## 3. Discussion

### 3.1. Structural Details

The structural parameters of **I** obtained by theoretical calculation agree closely with the experimental ones (X-ray diffraction). The atoms belonging to the S=C8-S-C7-C1′ chain are in a plane with experimental/theoretical dihedral angles S=C8-S-C7 and C8-S-C7-C1′ of −1.2°/2.65° and −174.7°/179.01°, respectively, and the same conformational preference (see Supplementary Materials, Table S1). The results show that the syn conformation is the unique form both in the gas phase (theoretical calculations) and solid-state (X-ray diffraction). The interatomic distance involving both sulfur atoms increases in the order C8=S > C8-S > C7-S, with the experimental values shorter than the theoretical ones. The thiocarbonyl distance is 1.644 Å (calc. 1.653 Å) and shows the weak double bond character due to inefficient p-overlapping. The two C-S single bond distances around the thioether function have substantial differences. The bond connecting the sulfur atom with the sp2-hybridized carbon atom, C8-S, is shorter (exp. 1.714(3) Å/calc. 1.758 Å) than the bond with the sp3-hybridized carbon atom, C7-S (exp. 1.807(4) Å/calc. 1.847 Å). These results indicate a significative charge delocalization from the thiocarbonyl sulfur to the thioether sulfur atom (S=C8-S- ↔ ^+^S-C8=^−^S-).

The arrangement of both aromatic rings around the chain connecting them shows differences when the theoretical calculation is compared with the experimental results. Calculations place the phenyl ring almost perpendicular to the carbodithioate plane with a S-C7-C1′-C2′ dihedral angle of 91.42° (exp: 62.9°), whereas the nitrophenyl ring is nearly coplanar to the chain in the crystal lattice (exp. 7.7°, calc. 36.25°). Additional calculations were performed with two molecules per unit cell in the crystalline structure, but no significant improvements were obtained. These results indicate the strong influence of the packing effects on molecular geometry.

### 3.2. Molecular Docking and Biological Test

Carbodithioates have been synthesized to test their antibacterial, antifungal, and antitumor activities [27,28,29]. The first studies with sulfur-containing compounds assayed against snake venom enzymes were substituted thiobenzoic acid S-benzyl esters [12], which showed an acceptable activity but had solubility drawbacks. Next, in order to improve the solubility, we synthesized and analyzed a group of thioesters derived from 2-Sulfenyl ethylacetate, but the bioactivity of these compounds was lower than substituted thiobenzoic acid S-benzyl esters [19].

Based on the above findings, one of the aims of this work was the synthesis of the thiocarbonyl analogue of the 4-nitrothiobenzoic acid benzyl ester (II), exchanging oxygen by sulfur, to study its ability to inhibit a myotoxic Asp-49 PLA_2_ from *C. durissus cumanensis* venom to complement the antivenom therapy. In this work, we showed, for the first time, the capacity of a carbodithioate to inhibit snake venom toxins. Owing to a higher reactivity and charge delocalization of the thiocarbonyl (I) compared to carbonyl (II) function, and considering the promising results obtained previously with II [12], greater activity of I was expected.

**Figure 6:**
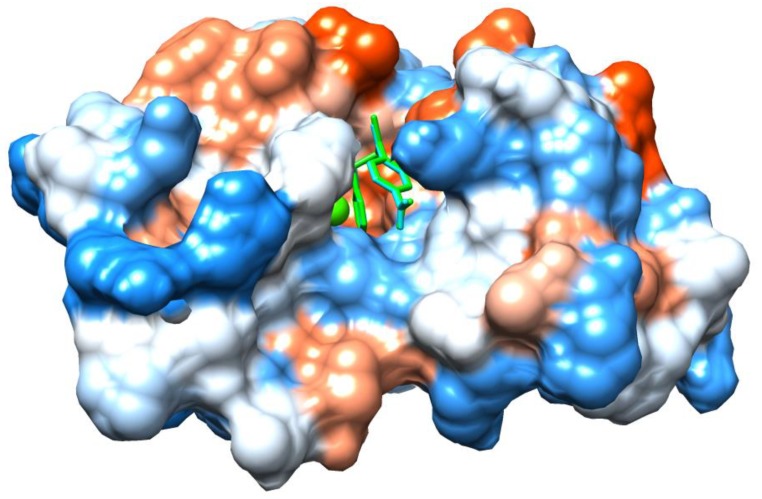
Docking conformations with highest affinities of **I** (green) and **II** (cyan) inside the hydrophobic channel of PLA_2._

Nevertheless, compounds I and II exhibited similar inhibitory capacity, around 50%, with a concentration of about 50 μM against PLA_2_ enzymatic activity. Docking results support the experimental findings because I and II showed the same interactions with the active site amino acids. The most stable conformations for the ligand–enzyme complexes are very similar and almost entirely aligned (see Figure 6), differing only in the observed distances of (C=S) and (C=O) bonds that are the only structural differences between these compounds. Furthermore, thiocarbonyl and carbonyl moieties did not show interaction with the active site amino acids. These findings explain why the theoretical affinities for compounds I and II are numerically equal, −7.1 kcal/mol. 

Instead, docking results suggested several favorable interactions between compounds I and II and the studied PLA_2_; the hydrogen bond found between the donor Nδ1 of His48 and one oxygen atom of the nitro group as the acceptor is a critical interaction to explain the biological activity of both compounds, since His48 is one of the catalytic residues (Figure 4).

Interestingly, the benzyl ring in both compounds participates simultaneously with two types of π interactions, one on each side. It showed a π-π T-shaped interaction with the Phe5 phenyl ring on one side, and a π-sulfur interaction with the sulfur atom of Cys45 on the other side. The former contact involves Phe5, a critical amino acid that belongs to the hydrophobic channel that allows the entrance of the substrate to the active site. π-sulfur contact, in conjunction with several Van der Waals interactions, could contribute to reinforcing the stabilization of the ligand–enzyme complex.

PLA_2_ are subdivided into catalytically active (Asp49) PLA_2_, and catalytically inactive PLA_2_ homologues. Recently, Salvador et al. reported the crystal structure of a complex formed by a catalytically inactive PLA_2_ (MjTX-II from *Bothrops moojeni*) and a synthetic inhibitor called Varespladib. The complex showed the presence of the inhibitor in the hydrophobic channel of the toxin, interacting particularly with His48 and Lys49 residues [30]. We hypothesize that compounds I and II may have a similar inhibitory mechanism on the catalytically inactive PLA_2_ since docking results suggest that these compounds interact with amino acids located at the hydrophobic channel of the toxin. Hence, compounds I and II could be classified as class I inhibitors of the homologous (Lys49) PLA_2_ [30]. The activity of these compounds could be due to the blocking of the hydrophobic channel and prevention of the binding of the fatty acid necessary for the toxin allosteric activation. Nevertheless, this should be confirmed by structural studies, and it is important to conduct cytotoxicity and myotoxicity studies.

## 4. Materials and Methods

### 4.1. Chemistry

Solvents were evaporated from solutions in a rotary evaporator Heidolph Laborota 4010 (Heidolph Instruments GmbH & CO. KG, Schwabach, Bayern, Germany) equipped with a ROTAVAP valve control. Melting points (m.p.) were recorded in a Melting point apparatus 9100 (Electrothermal, Stone, Staffordshire, England) and are not corrected. FTIR spectrum was measured between 4000 and 400 cm^−1^ (4 cm^−1^ resolution) in KBr pellets in a Spectrum Bx apparatus (Perkin Elmer Waltham, MA, USA). NMR spectra were measured in CDCl_3_ at 298 K on in Ascend 600 spectrometer (Bruker, Billerica, MA, USA). Chemical shifts, δ, are given in ppm relative to TMS (*δ* = 0 ppm) and are referenced by using the residual undeuterated solvent signal. Coupling constants, J, are reported in Hz, multiplicities being marked as singlet (s), and multiplet (m). In AA’BB’ system, coupling constants were calculated using algebraic methods [31,32,33].

The preparation of I was carried out on a dry flask. A mixture of 1 mmol of 4-nitrothiobenzoic acid benzyl ester, one mmol of 2,4-bis(4-phenoxyphenyl)-1,3,2,4-dithiadiphosphetane 2,4-disulfide, and 2 mL of anhydrous dimethoxyethane was heated for 8 h in an oil bath at 80˚C. The reaction mixture was cooled at room temperature. The oxygen–sulfur exchange reagent was filtered off, and the solvent phase was collected and evaporated. The crude product was purified by flash chromatography (ether/hexane 19:1). After evaporation of the solvent, red crystals were obtained. The reagent used for oxygen-sulfur exchange, 2,4-bis(4-phenoxyphenyl)-1,3,2,4-dithiadiphosphetane 2,4-disulfide, was obtained from P_2_S_5_ and diphenyl ether [25].

Red crystals, 85% yield (crude product) m.p. 68.9–69.1 °C. ^1^H NMR (CDCl_3_, 600 MHz 25 °C): *δ* = 8.13 (2H, AA’BB’system, *J* = 9, 2 and <1 Hz, H3 and H5); 7.98 (2H, AA’BB’system, *J* = 9, 2 and <1 Hz, H2 and H6); 7.35–7.15 (5H, m, C_6_H_5_); 4.51 (2H, s, CH_2_) ppm; ^13^C NMR (CDCl_3_, 62.98 MHz, 25 °C): *δ* = 224.4 (C8); 149.6 (C4); 148.85 (C1); 134.1 (C1′); 129.3 (C2 and C6); 128.8 (C2′ and C6′); 128.05 (C4′); 127.7 (C3′ and C5′); 123.6 (C4); 42.7 (C7) ppm. For atom numbering see Figure 1.

### 4.2. Quantum Chemical Calculations

Compounds I and II were built using Gauss View 5 [34] using the parameters of the experimental structure of I that was collected from The Cambridge Crystallographic Data Centre (Cambridge, UK) (CCDC 928055) [22]. The geometric parameters for the more stable conformers were calculated at the B3LYP/6-31+G (d,p) level of approximation using Gaussian 09 (Gaussian Inc, Wallingford, CT, USA) [26] program package, implemented on a personal computer. The optimized conformers were used as ligands for the docking study.

### 4.3. Molecular Docking

Molecular docking was carried out on a personal computer using Autodock Vina (Scripps Research Institute, San Diego, CA, USA) [35]. The structure of the PLA_2_ (PDB code 2QOG) from *Crotalus durissus terrificus* that showed 57% of identity with the PLA_2_ from *C. d. cumanensis* [36] was used in this study.

The protein structure was prepared using the Protein Preparation module implemented in the Maestro program and uploaded without water molecules. Hydrogen atoms were automatically added to the protein according to the chemical nature of each amino acid based on the ionized form expected in physiological conditions. This module also controls the atomic charges assignment. The 3D structure of the protein was relaxed through constrained local minimization, using the OPLS (Optimized Potentials for Liquid Simulations) force fields in order to remove possible structural mismatches due to the automatic procedure employed to add the hydrogen atoms. When necessary, bonds, bond orders, hybridizations, and hydrogen atoms were added, charges were assigned (a formal charge of +2 for Ca ion), and flexible torsions of ligands were detected.

To perform molecular docking experiments of compounds I–II with PLA_2_, the calcium ion was used as the center of the grid (*X* = 61.179, *Y* = 42.712 and *Z* = 47.465), whose size was 24 Å^3^. Exhaustiveness = 20. Finally, the ligand poses with the best affinity were chosen, and a visual inspection of the interactions at the active site was performed and recorded using UCSF Chimera (University of California, San Francisco, Ca, USA) (www.cgl.ucsf.edu/chimera/) [37]. Physicochemical properties with importance in oral bioavailability for compounds I and II were calculated using Molinspiration (Molinspiration, Nova ulica, Slovak Republic) [38].

### 4.4. Toxin Isolation

*Crotalus durissus cumanensis* venom was obtained from four specimens from Meta, in the southeast region of Colombia, and kept in captivity (Serpentarium of the Universidad de Antioquia, Medellín, Colombia). PLA_2_ was purified by reverse-phase HPLC on C-18 column eluted at 2.0 mL/min with a gradient from 0 to 100% acetonitrile in 0.1% trifluoroacetic acid (*v*/*v*). Absorbance in effluent solution was recorded at 280 nm [39].

### 4.5. Inhibition of Phospholipase A_2_ Activity

The measurements of enzymatic activity using the linear substrate 4N3OBA were performed according to the method described by Holzer and Mackessy [40] and adapted for a 96-well ELISA plate. The standard assay contained 200 μL of buffer (10 mM Tris–HCl, 10 mM CaCl_2_, 100 mM NaCl, pH 8.0), 20 μL of 10 mM of substrate (4NO3BA), 20 μL of sample (20 μg PLA_2_ or 20 μg PLA_2_ + several concentrations of 1) and 20 μL of water. The negative control was buffer. The inhibitory effect of 1 on PLA_2_ activity was determined through the co-incubation of the enzyme with 1 for 30 min at 37 °C. After the incubation period, the sample was added to the assay, and the reaction was monitored at 425 nm for 40 min (at 10 min intervals) at 37 °C. The quantity of chromophore released (4-nitro-3-hydroxy benzoic acid) was proportional to the enzymatic activity. The IC_50_ value was determined from the linear portion of the response-dose curve.

## 5. Conclusions

We demonstrated that the analogous compounds, benzyl 4-nitrobenzenecarbodithioate (I) and 4-nitrothiobenzoic acid S-benzyl ester (II), exhibit similar inhibitory capacity on myotoxic Asp49-PLA_2_ isolated from *C. durissus cumanensis* venom. The catalytic activity was reduced by about 50% using a concentration near to 50 μM for both compounds. These results show that the exchange of oxygen for sulfur to transform the carbonyl C (O) function into thiocarbonyl C (S) implies some structural differences that do not significantly alter the biological activity of the compound against a myotoxic PLA_2_.

Molecular docking studies for compounds I and II as ligands suggested the same way of binding to the active site of the enzyme. These interactions could explain the inhibition of enzymatic activity by blocking the normal progression of the catalytic cycle and by impeding the normal attachment of the substrate to the active site of the PLA_2_. The inhibition of the enzymatic activity of the myotoxic PLA_2_ showed similar results for I and II (between the experimental error), agreeing with the molecular docking findings. These results prompt us to use molecular docking as a reliable and economical tool to evaluate in silico the potential of PLA_2_ inhibitors.

We hope to expand the number of sulfur compounds with inhibition capacity against PLA_2_ and perform cytotoxicity studies and inhibition tests on homologous PLA_2_, as well as inhibition studies in animal models. Sulfur compounds could be valuable in search of potent inhibitors of snake venom enzymes.

## Figures and Tables

**Table 1:** Studied compounds and physicochemical properties.

Compound	X	MW ^a^	nON ^a^	nOHNH ^a^	LogP(calc) ^a^
I	S	289.38	3	0	4.41
II	O	273.31	4	0	3.86

^a^ Physicochemical properties calculated using Molinspiration. MW: Molecular mass. nON: Hydrogen bonds acceptor. nOHNH: Hydrogen bonds donator. LogP: Calculated octanol/water partition coefficient.

**Table 2:** Selected calculated (B3LYP/6-31+G(d,p)) and experimental IR frequencies (cm^−1^), relative intensities, and tentative assignments of the main fundamental vibrational modes of I.

Calculated ^a^	IR ^a^	Assignment ^b^
1647 m	1561 w	δi.p dithiobenzoate ring+νas NO_2_
1589 s	1516 vs	νas NO_2_
1382 vs	1343 vs	νs NO_2_
1250 m	1210 m	νC8-C1
1065 s	1048 vs	νC8=S
902 w	895 w	νas S-C8-C1
852 m	848 m	Scissor NO_2_
758 vw	752 w	o.o.p. wag NO_2_

^a^ Intensities have been classified semi-quantitatively in terms of very strong (vs), strong (s), medium (m), weak (w), and very weak (vw).^b^ ν, stretching; s, symmetric; as, antisymmetric; o.o.p out of plane.

## Supplementary Materials

The following are available online, Table S1: Homologous experimental and calculated (B3LYP/6-31+G(d,p)) intra-molecular bond distances (Å) and angles (°) of Benzyl 4-nitrobenzenecarbodithioate (I)., Figure S1: ^1^H NMR Spectrum of I Benzyl 4-nitrobenzenecarbodithioate, crude product., Figure S2: ^13^C NMR Spectrum of I Benzyl 4-nitrobenzenecarbodithioate, crude product., Figure S3: IR Benzyl 4-nitrobenzenecarbodithioate (I).
