# Supplementary material for: Sulfur Compounds as Inhibitors of Enzymatic Activity of a Snake Venom Phospholipase A2: Benzyl 4-nitrobenzenecarbodithioate as a Case of Study
Source: Molecules. 2020 Mar 18;25(6):1373. doi: 10.3390/molecules25061373 (PMC7144397; doi:10.3390/molecules25061373)
Supplement: Supplementary file 1 [file molecules-25-01373-s001.pdf]

Supplementary Material

Sulfur Compounds As Inhibitors of Enzymatic Activity of A Snake Venom Phospholipase A_2: Benzyl 4-nitrobenzenecarbodithioate As A Case of Study

Isabel Henao Castañeda [1,*], Jaime Andrés Pereañez [2], Lina María Preciado [2] and Jorge Jios [3,4]

1 Grupo de Investigación en Productos Naturales Marinos, Departamento de Farmacia, Facultad de Ciencias Farmacéuticas y Alimentarias, Universidad de Antioquia UdeA, Calle 70 No. 52–21, 050010 Medellín, Colombia

2 Programa de Ofidismo/Escorpionismo, Departamento de Farmacia, Facultad de Ciencias Farmacéuticas y Alimentarias, Universidad de Antioquia UdeA, Calle 70 No. 52–21, 050010 Medellín, Colombia.; andrespj20@gmail.com (J.A.P.); linampr@gmail.com (L.M.P.)

3 Laboratorio UPL (Unidad PLAPIMU-LASEISIC), Campus Tecnológico Gonnet (CIC-BA), Cno. Centenario e/505 y 508, 1897 Gonnet, República Argentina; jljios@quimica.unlp.edu.ar

4 Departamento de Química, Facultad de Ciencias Exactas, Universidad Nacional de La Plata, 47 esq. 115, 1900 La Plata, República Argentina

* Correspondence: isabel.henao@udea.edu.co; Tel.: +57-4-2195476

Figure S1. ^{1}H NMR Spectrum Benzyl 4-nitrobenzenecarbodithioate (I).

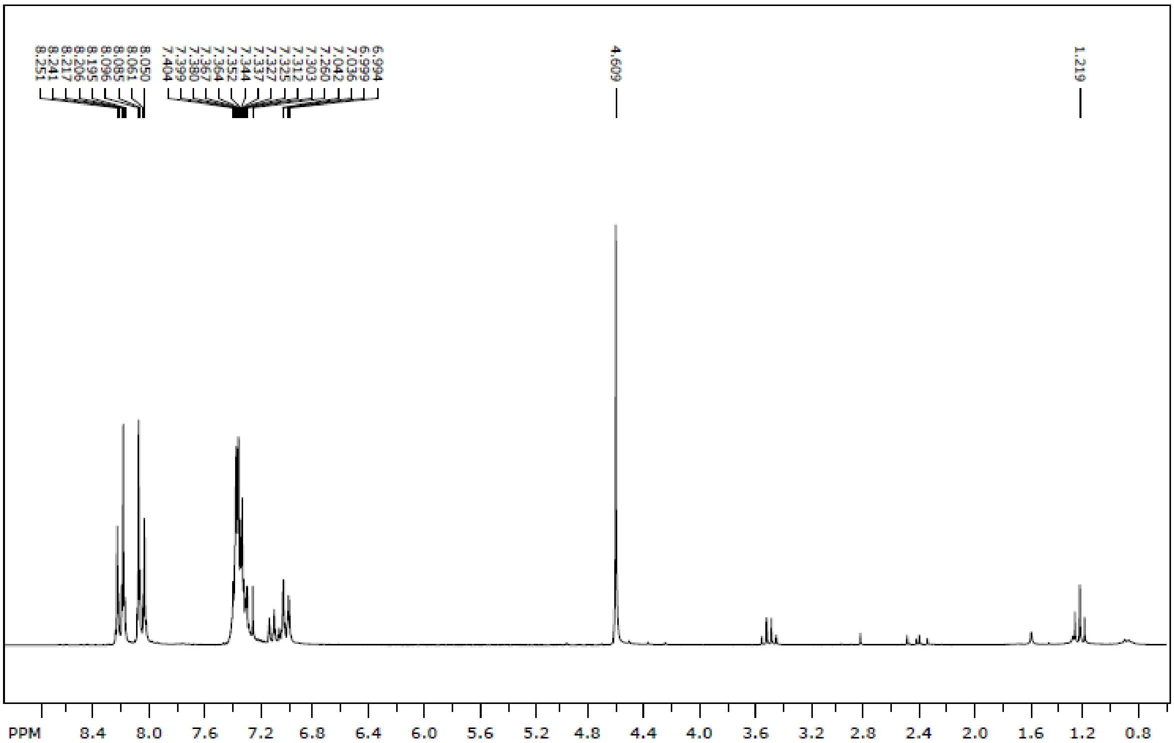

Figure S2. ^{13}C NMR Spectrum Benzyl 4-nitrobenzenecarbodithioate (I).

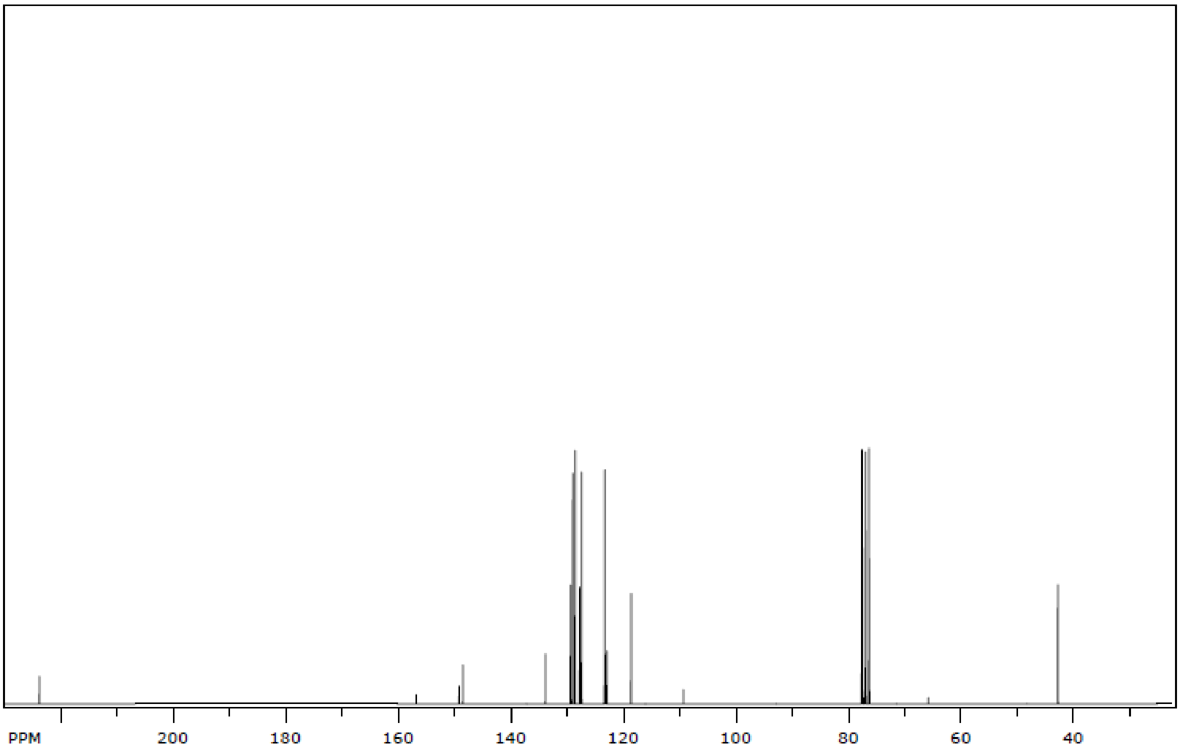

Figure S3. IR Benzyl 4-nitrobenzenecarbodithioate (I).

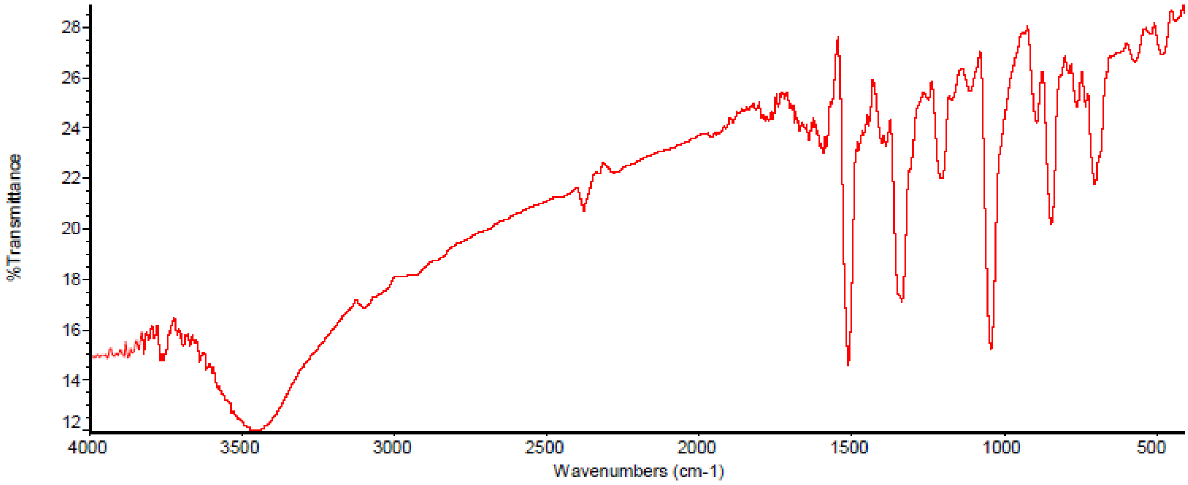

Table S1. Homologous experimental and calculated (B3LYP/6-31+G(d,p)) intra-molecular bond distances (Å) and angles (°) of Benzyl 4-nitrobenzenecarbodithioate.

**Bond Distances Å**	**Exp.**	**Calc.**
C1-C2	1.395(4)	1.408
C1-C6	1.399(4)	1.409
C1-C7	1.494(4)	1.494
C2-C3	1.370(5)	1.390
C3-C4	1.372(5)	1.396
C4-C5	1.371(4)	1.394
C4-N1	1.472(4)	1.472
C5-C6	1.374(5)	1.392
C7-S2	1.644(3)	1.653
C7-S1	1.714(3)	1.758
C8-C9	1.506(4)	1.508
C8-S1	1.807(4)	1.847
C9-C14	1.374(5)	1.403
C9-C10	1.385(4)	1.403
C10-C11	1.371(5)	1.396
C11-C12	1.372(6)	1.398
C12-C13	1.356(6)	1.398
C13-C14	1.377(5)	1.397
N1-O1	1.216(4)	1.231
N1-O2	1.217(4)	1.235
**Bond Angles**		
C2-C1-C6	118.2(3)	118.89
C2-C1-C7	120.1(3)	119.50
C6-C1-C7	121.7(3)	121.61
C3-C2-C1	120.2(3)	120.90

	**Exp.**	**Calc.**
C2-C3-C4	119.9(3)	118.73
C3-C4-C5	121.8(3)	121.95
C3-C4-N1	119.0(3)	119.07
C5-C4-N1	119.2(3)	118.99
C4-C5-C6	118.3(3)	118.72
C5-C6-C1	121.5(3)	120.80
C1-C7-S2	122.9(2)	122.25
C1-C7-S1	114.0(2)	112.49
S2-C7-S1	123.1(2)	125.26
C9-C8-S1	107.9(2)	109.39
C14-C9-C10	118.4(3)	119.06
C14-C9-C8	121.0(3)	120.36
C10-C9-C8	120.6(3)	120.58
C11-C10-C9	120.5(3)	120.57
C12-C11-C10	119.8(4)	120.03
C13-C12-C11	120.5(4)	119.76
C12-C13-C14	119.8(4)	120.22
C9-C14-C13	121.0(3)	120.36
O1-N1-02	123.4(3)	124.36
O1-N1-C4	118.3(3)	117.96
O2-N1-C4	118.3(3)	117.68
C7-S1-C8	104.42(16)	103.84
**Torsion Angles**		
S2-C7-S1-C8	-1.2	2.65
S2-C7-C1-C2	7.7	36.25
S2-C7-C1-C6	-174.1	-143.64
C7-S1-C8-C9	-174.7	179.01
S1-C8-C9-C10	62.9	91.42
S1-C8-C9-C14	-118.4	-88.86
C8-C9-C10-C11	179.8	179.55
C8-C9-C14-C13	-180.0	-179.57
C9-C10-C11-C12	-0.5	0.14
